# Liraglutide combined with dapagliflozin treatment improves myocardial disease and endothelial dysfunction in T2DM mice

**DOI:** 10.1111/jdi.70363

**Published:** 2026-07-03

**Authors:** An‐Jun Tan, Tian‐Rong Li, Jing‐Jing Yang, Xiao‐Lu Li, Wen‐Qin Li, Jin‐Wen Yu

**Affiliations:** ^1^ Department of Geriatric Medicine The First People's Hospital of Yunnan Province, The Affiliated Hospital of Kunming University of Science and Technology Kunming China

**Keywords:** Dapagliflozin, Diabetic cardiomyopathy, Liraglutide

## Abstract

**Background:**

Diabetic cardiomyopathy (DCM) and endothelial dysfunction are major drivers of cardiovascular morbidity in type 2 diabetes (T2DM). The present study investigates whether combined therapy with liraglutide (LIRA) and dapagliflozin (DAPA) provides synergistic cardioprotection and vascular improvement in a murine model of T2DM.

**Methods:**

A murine model of T2DM was established by high‐fat diet feeding followed by low‐dose streptozotocin injection. Diabetic mice were treated for 4 weeks with LIRA, DAPA, or their combination. Echocardiographic analysis was performed to assess cardiac systolic function and structural parameters. Systemic metabolic parameters, cardiac injury biomarkers, histopathology, oxidative stress markers, and inflammation were assessed. Key signaling pathways were evaluated by Western blot and qPCR.

**Results:**

Compared with monotherapies, combined LIRA and DAPA treatment produced superior metabolic improvements, including attenuated weight loss and enhanced glucose tolerance. The combination also more effectively reduced markers of myocardial injury (CK‐MB, LDH), diminished cardiac fibrosis, and preserved aortic architecture while inhibiting endothelial apoptosis. Mechanistically, co‐administration synergistically activated both the antioxidative NRF2/HO‐1 pathway and the vasoprotective AMPK/PKA‐eNOS axis, leading to a more comprehensive suppression of oxidative stress, inflammation, and apoptosis in cardiac and vascular tissues.

**Conclusion:**

The combined use of LIRA and DAPA exerts synergistic cardioprotective and vasoprotective effects in a preclinical model of T2DM‐induced DCM. This synergistic effect may be mediated by the coordinated activation of the NRF2/HO‐1 and AMPK/PKA‐eNOS signaling pathways, leading to the attenuation of the key pathological drivers of diabetic cardiovascular injury. These findings provide a mechanistic rationale for the clinical exploration of GLP‐1RA and SGLT2i combination therapy in DCM.

## Graphical abstract

Liraglutide combined with Dapagliflozin treatment improves myocardial disease and endothelial dysfunction in T2DM mice. Diabetic cardiomyopathy (DCM) and endothelial dysfunction were established in mice via HFD and STZ injection. The combination therapy of Liraglutide and Dapagliflozin synergistically improved metabolic parameters and attenuated cardiac/vascular pathological changes compared to monotherapies. Specifically, the combination reduced myocardial injury (CK‐MB, LDH), cardiac fibrosis (Collagen I/III, TGF‐β1), and apoptosis in both heart and aorta. Furthermore, it suppressed oxidative stress and inflammation while activating protective signaling pathways. The synergistic effect could be partially attributed to the concurrent activation of the NRF2/HO‐1 and AMPK/PKA‐eNOS pathways.

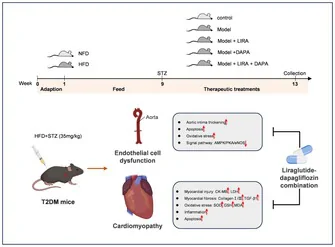

## INTRODUCTION

Type 2 diabetes mellitus (T2DM) is a metabolic disorder characterized by insulin resistance and/or impaired insulin secretion. As of 2019, it affected approximately 463 million people worldwide, with diabetes‐related disability‐adjusted life years (DALYs) reaching 66 million^1^. T2DM is not only defined by chronic hyperglycemia but also represents a systemic metabolic disturbance, commonly accompanied by dyslipidemia, chronic low‐grade inflammation, oxidative stress, endothelial dysfunction, and microvascular impairment^2^, ^3^. Consequently, T2DM has long been recognized as a major risk factor for cardiovascular disease (CVD)^4^.

Diabetic cardiomyopathy (DCM) refers to a distinct form of myocardial structural (e.g., left ventricular hypertrophy and myocardial fibrosis) and functional abnormalities (e.g., diastolic dysfunction) that occur in individuals with diabetes, independent of coronary artery disease, hypertension, valvular heart disease, or other cardiac conditions^4^, ^5^. The development of DCM is multifactorial, and among these pathogenic mechanisms, dysfunction of vascular endothelial cells, including the aorta and coronary arteries, is considered a “key bridge” between T2DM and cardiovascular complication^6^. Specifically, the metabolic disorders triggered by hyperglycemia lead to mitochondrial dysfunction and the activation of NADPH oxidase, both of which contribute to an overproduction of reactive oxygen species (ROS), which triggers oxidative stress and inflammation, reduces NO bioavailability by scavenging and inhibiting its synthesis, and consequently exacerbates endothelial dysfunction^7^. Concurrently, this endothelial dysfunction promotes the overactivity of the renin‐angiotensin‐aldosterone system and the sympathetic nervous system, leading to vasoconstriction, increased cardiac preload and afterload, and directly promoting myocardial hypertrophy, interstitial fibrosis, and ventricular remodeling^6^, ^8^. Therefore, endothelial dysfunction is an early and modifiable pathological process that persists throughout the development of DCM.

The incidence of DCM in diabetic patients is as high as 16.9%, closely associated with the high rates of heart failure (HF) and mortality^9^. However, there are currently no therapies available for DCM. Despite the capability of traditional glucose‐lowering treatments to effectively control blood glucose, these methods have not demonstrated benefits in reducing vascular complications and some may even elevate the risk of HF^10^, ^11^; nonetheless, the emergence of new glucose‐lowering drugs has introduced a renewed opportunity for cardiovascular risk management in diabetic patients. Dapagliflozin (DAPA), one of the earliest sodium‐glucose cotransporter 2 inhibitors (SGLT2i) used for blood glucose control in T2DM patients, reduces renal glucose reabsorption by blocking SGLT2, thereby lowering blood glucose levels^12^. Because this mechanism only works when blood glucose exceeds a reduced renal threshold, it helps minimize the occurrence of hypoglycemia. The DECLARE‐TIMI study demonstrated that among 17,160 T2DM patients with cardiovascular disease or risk factors, DAPA significantly reduced the composite endpoint of cardiovascular death or hospitalization due to HF^13^. Subsequent research further confirmed that DAPA could significantly decrease the risk of worsening HF and cardiovascular mortality, irrespective of ejection fraction status or diabetic diagnosis^14^, ^15^. Different from DAPA, liraglutide (LIRA) is a human glucagon‐like peptide‐1 receptor agonist (GLP‐1RA) that promotes insulin secretion and inhibits glucagon release in a glucose‐dependent manner by activating the GLP‐1R^16^. It also delays gastric emptying and increases satiety, thereby effectively controlling blood glucose levels^16^. The LEADER study demonstrated that LIRA significantly reduces the risk of cardiovascular death and all‐cause mortality in high‐risk T2DM patients, with additional benefits including weight loss and a low risk of hypoglycemia, indicating a favorable safety profile^17^. Therefore, both DAPA and LIRA have become novel and promising cardioprotective medications and are recommended for priority use in patients with T2DM who are at high risk of atherosclerotic cardiovascular disease or HF^18^, ^19^, ^20^. However, the mechanisms by which they exert their effects are not yet fully understood.

Given that DAPA and LIRA have distinct yet complementary mechanisms of action, demonstrated efficacy in reducing cardiovascular events and improving cardiorenal outcomes, and favorable safety profiles, their combination holds promise as a cornerstone of optimized therapeutic strategies for patients with T2DM or high cardiovascular risk^21^, ^22^. This study aims to evaluate and compare the protective effects of DAPA, LIRA, and their combination in DCM, with a focus on their modulation of endothelial dysfunction and underlying molecular mechanisms. By elucidating the differential and potentially synergistic actions of DAPA and LIRA, this work provides important insights for advancing personalized cardiometabolic therapy in diabetic and high‐risk populations.

## MATERIALS AND METHODS

### Animal

Eight‐week‐old C57BL/6J mice (*n* = 30) were obtained from Shanghai Baiken Biotechnology Co., Ltd., and housed with a 12‐h light/dark cycle, at 22 ± 1°C and 50% ± 10% humidity, with free access to food and water. All experimental procedures involving animals were approved by the Institutional Animal Care and Use Committee of the Kunming University of Science and Technology (KUST) (Animal protocol number: AP‐KUST‐202508020067).

The T2DM model was established according to prior studies^23^, ^24^. Briefly, mice were fed a high‐fat diet (HFD; 60% fat, 20% protein, 20% carbohydrate) for 9 weeks, followed by a single intraperitoneal injection of streptozotocin (STZ; 35 mg/kg, dissolved in sodium citrate buffer; Aladdin, S110910) after 15 h of fasting. Diabetes was considered successfully established when fasting blood glucose levels were ≥ 16.7 mmol/L and 2‐h postprandial glucose levels were < 11.1 mmol/L, as measured from tail vein blood using a Roche glucometer. Control mice were fed a standard chow diet (15% fat, 65% carbohydrate, 20% protein) and received intraperitoneal injection of an equal volume of sodium citrate buffer. The diabetic mice were then randomly divided into four groups and treated for 4 weeks. At approximately 9:00–10:00 am daily, the LIRA group (*n* = 6) received subcutaneous injections of liraglutide (25 nmol/kg/day), the DAPA group (*n* = 6) received oral administration of dapagliflozin (1 mg/kg/day), and the combination group (*n* = 6) received both liraglutide and dapagliflozin at the same doses^25^. The control (*n* = 6) and model groups (*n* = 6) were administered oral saline (0.9% NaCl) daily. Body weight was recorded weekly throughout the treatment period.

At the end of the 4‐week treatment period, cardiac function was evaluated using a small animal ultrasound imaging system (SiliconWave 30, Suzhou Cona Medical Co., Ltd., China) equipped with a 30‐MHz linear transducer. Mice were anesthetized with 1.5%–2% isoflurane and maintained on a heated stage to sustain body temperature. Two‐dimensional (2D) images of the left ventricle were acquired in the parasternal long‐axis view. M‐mode echocardiography was then performed at the level of the papillary muscles to quantify left ventricular function. Left ventricular ejection fraction (EF) was calculated automatically using the software (Skyline Ultrasound Imaging Software V1.0.1, Cona Medical).

An IPGTT was performed following the completion of the 4‐week intervention. On the day prior to the experiment, a glucose solution (Aladdin, D274366) was equilibrated to room temperature overnight. Mice were fasted for 15 h, and glucose was injected intraperitoneally at a dose of 2 g/kg body weight. Tail vein blood was collected at predetermined time points (0, 30, 60, and 120 min post‐injection) for determination of blood glucose concentrations.

At the end of the experiment, mice were euthanized, and the thoracic cavity was opened. The heart was rapidly excised, rinsed with ice‐cold phosphate‐buffered saline (PBS, 0.01 M, pH 7.4), blotted dry, and weighed. The thoracoabdominal aorta was carefully dissected and rinsed with ice‐cold PBS. A portion of the heart and aorta was immediately snap‐frozen in liquid nitrogen for subsequent molecular analyses, and another portion was fixed in 4% paraformaldehyde for paraffin embedding.

### Enzyme‐linked immunosorbent assay (ELISA)

ELISA was employed to measure myocardial injury markers—creatine kinase‐MB (CK‐MB, H197‐1‐1, Nanjing Jiancheng Bioengineering Institute) and lactate dehydrogenase (LDH, A020‐2‐2, Nanjing Jiancheng Bioengineering Institute)—as well as oxidative stress parameters, including malondialdehyde (MDA, S0131S, Beyotime Biotechnology), reduced glutathione (GSH, ml076450, MLBIO), and superoxide dismutase (SOD, ml092620, MLBIO) in mouse cardiac tissues. Mouse heart tissues were homogenized in physiological saline using an electric ultrasonic homogenizer. The homogenates were centrifuged at 4,000 × *g* for 15 min at 4°C, and the supernatants were collected for analysis. Absorbance was measured at 450 nm with a microplate reader. Concentrations were calculated based on standard curves and normalized to total protein content.

### Histochemical staining

Cardiac and aortic tissues were stained with hematoxylin and eosin (H&E; Beyotime, C0105S) for histological examination. Deparaffinized tissue sections were immersed in hematoxylin solution for 4 min, differentiated for 20 s, and then counterstained with eosin for 2 min.

Masson's trichrome staining was performed to assess myocardial fibrosis in mouse heart tissue using a Solarbio staining kit (G1346) according to the manufacturer's instructions. Briefly, sections were stained with ponceau‐fuchsin solution for 7 min, treated with phosphomolybdic acid solution for 1 min, and then stained with aniline blue for 1 min 30 s. The stained sections were mounted with neutral balsam and observed under a microscope. Neutral balsam was used for mounting, and the sections were observed under a microscope.

Sirius Red staining was performed to evaluate collagen deposition and myocardial fibrosis in mouse heart tissue. Briefly, deparaffinized tissue sections were stained with Sirius Red solution (Servicebio, G1078) for 60 min at room temperature. After washing in distilled water, the sections were rapidly dehydrated in absolute alcohol, cleared in xylene, and mounted with neutral balsam. The stained sections were observed under a light microscope to assess the distribution of collagen fibers, which appeared red against a pale yellow or unstained background.

### Quantitative PCR (qPCR)

The cardiac tissue was homogenized in Trizol Reagent (15596026, Invitrogen), and RNA was isolated using the standard chloroform‐isopropanol precipitation method. For cDNA synthesis, 1 μg of total RNA was reverse‐transcribed using a reverse transcription kit (R333‐01, Vazyme) at 50°C for 15 min. Subsequently, qPCR was performed using the synthesized cDNA as a template on a qTOWER 3G real‐time PCR system (Analytik Jena, Germany) with SYBR Green qPCR Master Mix (RR820A, Takara). The primers used in this study for qPCR analysis were as follows: GAPDH (forward: TGGTGAAGCAGGCATCTGAG, reverse: TGAAGTCGCAGGAGACAACC), collagen I (forward: AGCACGTCTGGTTTGGAGAG, reverse: GACATTAGGCGCAGGAAGGT), collagen III (forward: GAGGAATGGGTGGCTATCCG, reverse: TCGTCCAGGTCTTCCTGACT), and TGF‐β1 (forward: ACTGGAGTTGTACGGCAGTG, reverse: GGGCTGATCCCGTTGATTTC). Gene expression levels were normalized to GAPDH expression, and relative mRNA expression was calculated using the 2^−∆∆Ct^ method.

### Western blotting

Cardiac and aortic tissues were homogenized in ice‐cold RIPA buffer supplemented with phenylmethylsulfonyl fluoride (PMSF; ST2573, Beyotime). Lysates were centrifuged at 4°C, and the supernatants were collected for protein quantification (BCA assay kit, P0010, Beyotime). Protein samples were denatured and then separated by SDS‐PAGE on 12% polyacrylamide gels, followed by transfer onto nitrocellulose membranes (66,485, Pall). Membranes were blocked with 5% (w/v) non‐fat milk (A600669‐0250, Sangon Biotech) in TBST for 1.5 h at room temperature and then incubated overnight at 4°C with the following primary antibodies at specified dilutions: TNF‐α (1:1,000, 17590‐1‐AP, Proteintech), IL‐1β (1:1,000, AF5103, Affinity), IL‐6 (1:500, GB11117, Servicebio), Bax (1:8,000, 50599‐2‐Ig, Proteintech), BCl‐2 (1:1,000, AF6139, Affinity), cleaved caspase‐3 (1:1,000, AF7022, Affinity), 3‐NT (1:1,000, HY‐P81216, MCE), NRF2 (1:1,000, BF8017, Affinity), HO‐1 (1:20,000, 10,701‐1‐AP, Proteintech), p‐AMPK (1:1,000, AF3423, Affinity), AMPK (1:1,000, AF6423, Affinity), PKA (1:1,000, AF7746, Affinity), p‐eNOS (1:1,000, AF3247, Affinity), and eNOS (1:20,000, 27120‐1‐AP, Proteintech). After extensive washing, the membranes were incubated with horseradish peroxidase (HRP)‐conjugated secondary antibodies (S0001/S0002, Affinity) for 1 h at room temperature. Protein bands were visualized using an ECL reagent under a multifunctional imaging system, and band densities were quantified with ImageJ software. Protein expression levels were normalized to GAPDH (AB0037, Abways).

### Terminal deoxynucleotidyl transferase dUTP nick end labeling (TUNEL) assay

To detect apoptotic cells in aortic tissue sections, a TUNEL assay was performed according to the manufacturer's instructions (Roche). Briefly, tissue sections were treated with Proteinase K working solution at 37°C for 15 min. After equilibration with TdT buffer (37°C, 30 min), samples were incubated with TUNEL labeling mixture at 37°C for 60 min in the dark. Following DAPI counterstaining, fluorescent signals were imaged using a fluorescence microscope, and TUNEL‐positive cells (green fluorescence) were counted in five random fields per section.

### Immunofluorescence staining

Immunofluorescence staining was performed on aortic tissue sections. After permeabilization with 0.2% Triton X‐100 in PBS at 37°C for 10 min, sections were blocked with 5% BSA for 30 min at room temperature. Primary antibodies against CD31 (1:300, AF6191, Affinity) and 3‐NT (1:150, HY‐P81216, MCE) were applied and incubated overnight at 4°C. After washing, Alexa Fluor‐conjugated secondary antibodies were used for detection in the dark. Following DAPI counterstaining, fluorescent signals were imaged using a fluorescence microscope. The number of CD31‐positive cells as well as 3‐NT was counted in five random fields per section.

### Statistical analysis

Data are presented as mean ± standard deviation (SD) from at least three independent experiments. Statistical analyses were performed using GraphPad Prism software. Normality of distribution was assessed using the Shapiro–Wilk test, and homogeneity of variances was confirmed by Levene's test. For comparisons among three or more groups, one‐way analysis of variance (anova) was performed, followed by Tukey's honestly significant difference (HSD) post hoc test for multiple comparisons. A P value of less than 0.05 was considered statistically significant.

## RESULTS

### Liraglutide and dapagliflozin ameliorate metabolic disturbances and attenuate myocardial injury in a murine model of type 2 diabetic cardiomyopathy

**Figure 1:**
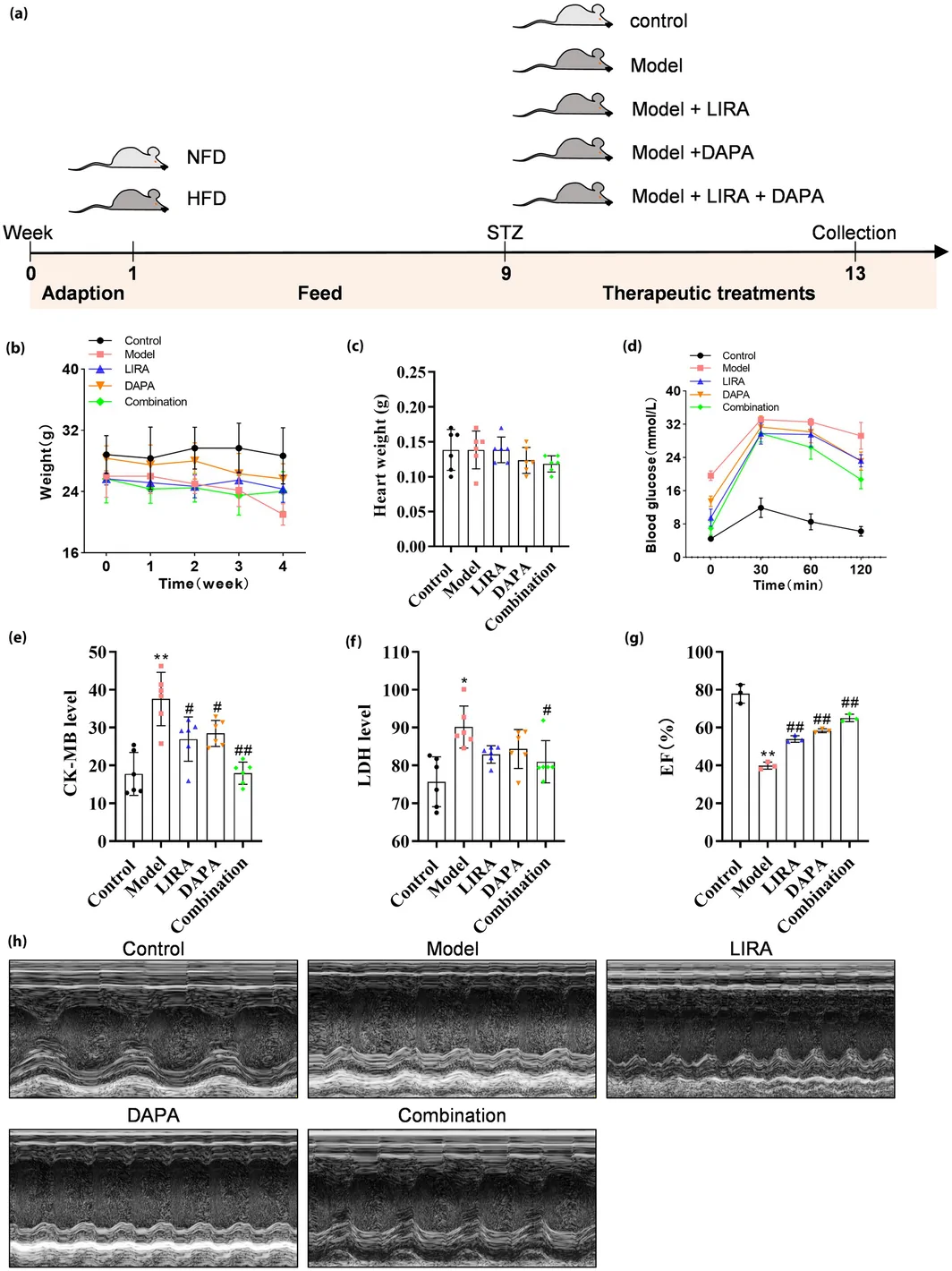
Liraglutide and dapagliflozin improve systemic metabolism and reduce myocardial injury in a murine model of type 2 diabetic cardiomyopathy. (a) Experimental timeline and group assignment: mice were fed a high‐fat diet (HFD) for 8 weeks followed by low‐dose streptozotocin (STZ) to induce diabetes, then treated with vehicle, liraglutide, dapagliflozin, or their combination for 4 weeks (*n* = 6 per group). (b) Body weight trajectories during HFD feeding and intervention. (c) Absolute heart weight at endpoint. (d) Intraperitoneal glucose tolerance test (IPGTT; 2 g/kg glucose, i.p.) performed after 4 weeks of treatment; data expressed as blood glucose concentration over time. (e, f) Serum levels of cardiac injury biomarkers measured by ELISA: creatine kinase‐MB (CK‐MB, e), and lactate dehydrogenase (LDH, f). (g) Statistical analysis of left ventricular ejection fraction (EF). (h) Representative M‐mode echocardiographic tracings showing left ventricular systolic function. Data are presented as mean ± SD, *n* = 6. *P < 0.05, **P < 0.01 vs. control; ^#^P < 0.05, ^##^P < 0.01 vs. model.

To evaluate the cardiometabolic protective effects of LIRA and DAPA—administered as monotherapies or in combination—in a murine model of T2DM‐induced cardiomyopathy, we induced T2DM in C57BL/6J mice through HFD feeding followed by low‐dose STZ administration. As detailed in the experimental timeline (Figure 1a), animals were randomly allocated into five groups: a non‐diabetic control group, a T2DM model group, and three therapeutic cohorts within the T2DM population receiving liraglutide, dapagliflozin, or their combination, respectively. All interventions were maintained for four consecutive weeks. Compared to the normal group, mice in the T2DM model group experienced a significant reduction in body weight. However, administration of liraglutide, dapagliflozin, or their combination during the treatment period mitigated weight loss in T2DM mice relative to the untreated model group, with the combination therapy exhibiting the greatest protective effect (Figure 1b). Relative to the model group, the dapagliflozin monotherapy and combination therapy cohorts demonstrated a notable decrease in heart weight (Figure 1c). Systemic glucose homeostasis was evaluated using an IPGTT. Mice with T2DM demonstrated marked glucose intolerance, evidenced by persistent hyperglycemia following administration of an intraperitoneal glucose bolus (2 g/kg). Both LIRA and DAPA monotherapies resulted in significant enhancement of glucose clearance relative to the model group, with the combination therapy yielding the most pronounced improvement (Figure 1d). To quantify myocardial injury, levels of cardiac damage biomarkers were measured by ELISA. The model group exhibited significant increases in alanine CK‐MB (Figure 1e) and LDH (Figure 1f), indicative of pronounced cardiomyocyte stress and compromised membrane integrity. Both DAPA treatment and LIRA administration significantly reduced CK‐MB levels. Notably, combined DAPA and LIRA therapy produced the most pronounced cardioprotective effect, lowering both biomarkers to concentrations comparable to those observed in non‐diabetic control subjects. Furthermore, echocardiographic analysis revealed that the T2DM model group displayed significantly reduced ejection fraction compared to the control group, indicating impaired cardiac systolic function (Figure 1g). Treatment with either LIRA or DAPA alone resulted in partial restoration of EF, while the combination therapy fully normalized EF to levels comparable to the non‐diabetic control group. Consistent with these functional improvements, representative M‐mode echocardiographic images (Figure 1h) demonstrated that the combination therapy effectively preserved left ventricular wall thickness and contractility, ameliorating the structural and functional deterioration observed in the T2DM model group. In summary, these data demonstrate that LIRA and DAPA—particularly when co‐administered—effectively mitigate obesity‐associated metabolic dysfunction, improve systemic glucose tolerance, and attenuate myocardial injury in a murine model of T2DM‐induced cardiomyopathy.

### Combined therapy synergistically suppresses cardiac fibrosis and downregulates profibrotic gene expression in diabetic hearts

**Figure 2:**
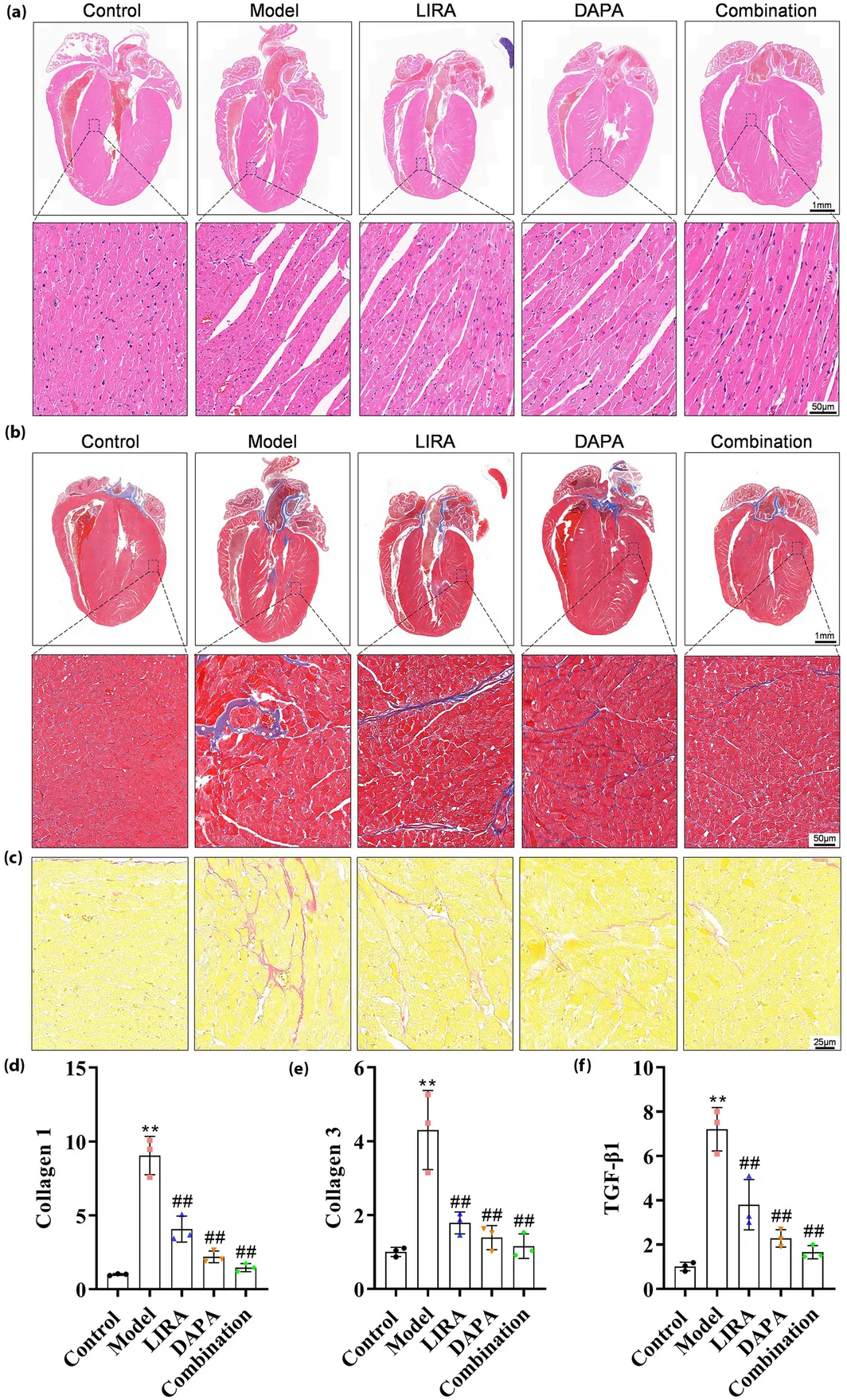
Combined liraglutide and dapagliflozin therapy attenuates cardiac fibrosis and suppresses profibrotic signaling in diabetic hearts. (a) Representative hematoxylin and eosin (H&E) staining of left ventricular sections showing myocardial architecture (scale bar: 50 μm). (b) Masson's trichrome staining illustrating interstitial and perivascular collagen deposition (blue); scale bar: 50 μm. (c) Representative images of Picrosirius Red staining viewed under polarized light to visualize collagen deposition (red/yellow birefringence); scale bar: 100 μm. (d–f) mRNA expression levels of Collagen I (d), Collagen III (e), and TGF‐β1 (f) in cardiac tissue. *n* = 3, Data are presented as mean ± SD, *n* = 3.  **P < 0.01 vs. control;  ^##^P < 0.01 vs. model.

Subsequently, histopathological examination of cardiac tissue revealed significant structural alterations in the T2DM model group compared with the control. H&E staining demonstrated marked myocyte disarray, interstitial widening, and focal inflammatory cell infiltration in diabetic hearts, consistent with pathological cardiomyopathy (Figure 2a). In contrast, both LIRA and DAPA monotherapies partially restored myocardial architecture, while the combination treatment yielded the most pronounced improvement, with near‐normalization of myofiber alignment and reduced interstitial space. Masson's trichrome staining further confirmed the presence of extensive collagen deposition in the model group, indicative of myocardial fibrosis (Figure 2b). The fibrotic areas were prominently localized in the interstitium and perivascular regions. Treatment with either LIRA or DAPA significantly attenuated fibrosis, as evidenced by reduced blue‐stained collagen fibers. Notably, the combination therapy produced a synergistic anti‐fibrotic effect, resulting in minimal fibrotic lesions comparable to those observed in the control group. Consistent with the histological findings, Picrosirius Red staining confirmed the amelioration of collagen accumulation following treatment (Figure 2c). At the molecular level, qPCR analysis revealed that the diabetic hearts exhibited a marked upregulation of key fibrotic transcripts—Col1a1, Col3a1, and Tgfb1—which was significantly suppressed by both LIRA and DAPA monotherapies, with their combination providing the most potent reversal of this pro‐fibrotic gene expression (Figure 2d–f). Collectively, these findings demonstrate that LIRA and DAPA, particularly in combination, exert potent anti‐fibrotic effects in T2DM‐induced cardiomyopathy, as evidenced by improved histomorphology, reduced collagen deposition, and downregulation of fibrotic gene expression.

### Combined administration mitigates oxidative stress, inflammation, and apoptosis in the diabetic myocardium

**Figure 3:**
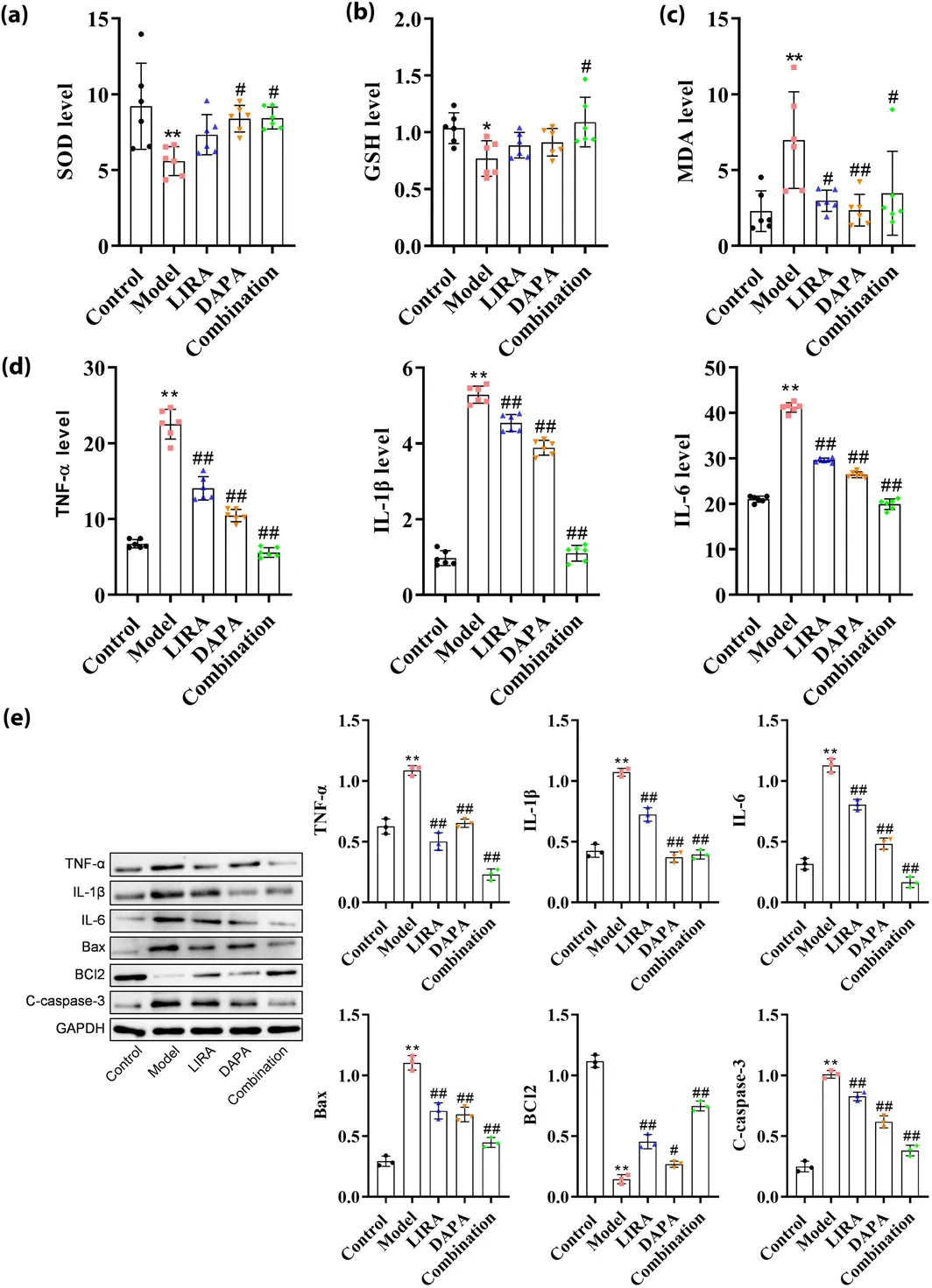
Liraglutide and dapagliflozin alleviate oxidative stress, inflammation, and apoptosis in the diabetic myocardium. (a–c) Biochemical assays of oxidative stress markers in heart homogenates: Superoxide dismutase (SOD) activity (a), reduced glutathione (GSH) content (b), and malondialdehyde (MDA) levels (c), *n* = 6. (d) Serum levels of pro‐inflammatory cytokines (TNF‐α, IL‐1β, and IL‐6) measured by ELISA, *n* = 6. (e) Western blot analysis of pro‐inflammatory cytokines (TNF‐α, IL‐1β, IL‐6) and apoptotic proteins (Bax, Bcl‐2, cleaved caspase‐3); GAPDH served as loading control, *n* = 3. Data are presented as mean ± SD. *P < 0.05, **P < 0.01 vs. control; ^#^ *P* < 0.05, ^##^ *P* < 0.01 vs. model.

The impact of LIRA and DAPA, alone or in combination, on oxidative stress, inflammation, and apoptosis in the hearts of T2DM mice, we measured key biomarkers at the molecular level. Diabetic hearts exhibited significantly impaired antioxidant capacity, with a marked decrease in SOD activity and GSH levels, while MDA—a marker of lipid peroxidation—was substantially elevated (Figure 3a–c). These findings indicate severe oxidative stress in the model group. Treatment with either LIRA or DAPA partially restored antioxidant defenses: SOD activity and GSH levels were increased, while MDA accumulation was attenuated. Notably, the combination therapy demonstrated superior efficacy, significantly enhancing SOD and GSH levels and further suppressing MDA production, suggesting a synergistic effect in mitigating cardiac oxidative damage.

Inflammatory responses and apoptosis in cardiac tissue were next evaluated via Western blot analysis. As determined by ELISA, the model group showed significantly elevated levels of pro‐inflammatory cytokines (TNF‐α, IL‐1β, and IL‐6) in the serum (Figure 3d). Consistent with these findings, cardiac tissue also showed pronounced upregulation of pro‐inflammatory cytokines, including TNF‐α, IL‐1β, and IL‐6 (Figure 3e). Both LIRA and DAPA significantly reduced the expression of these mediators, with the combination regimen achieving the most robust suppression. In parallel, markers of apoptosis were markedly altered in diabetic hearts. Bax expression was significantly increased, whereas anti‐apoptotic BCl‐2 was downregulated, leading to an elevated Bax/Bcl‐2 ratio. Additionally, cleaved caspase‐3, a key executioner of apoptosis, was markedly upregulated. All three apoptotic markers were effectively reversed by LIRA and DAPA treatment, with the combination therapy showing the greatest reduction in Bax and cleaved caspase‐3 levels and the most significant restoration of BCl‐2 expression. Collectively, these results demonstrate that LIRA and DAPA, particularly when administered in combination, exert potent antioxidant, anti‐inflammatory, and anti‐apoptotic effects in the hearts of T2DM mice, thereby alleviating multiple pathological mechanisms underlying DCM.

### Combined therapy inhibits endothelial apoptosis and ameliorates vascular dysfunction in diabetes

**Figure 4:**
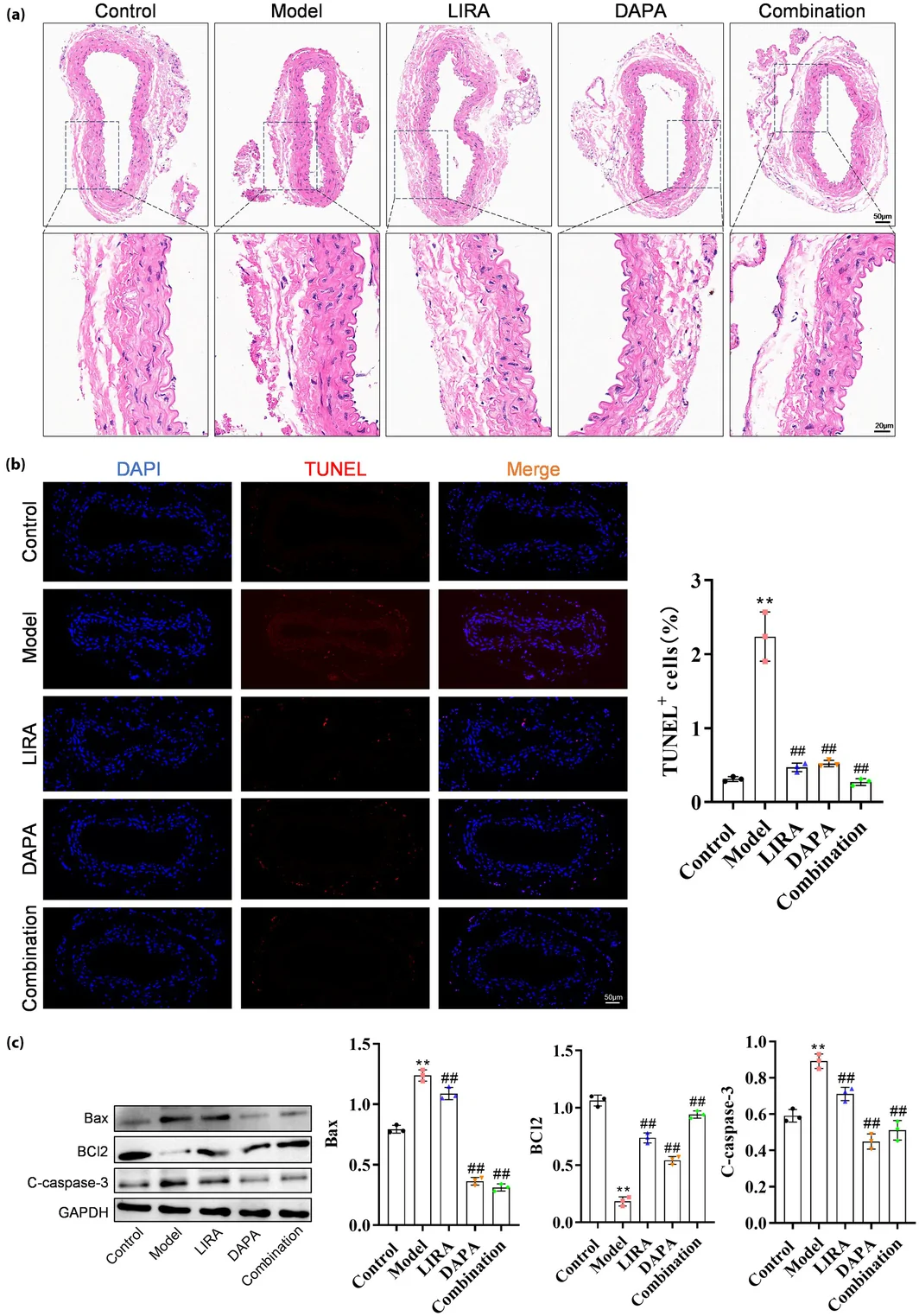
Combined therapy inhibits endothelial apoptosis and ameliorates vascular dysfunction in diabetes. (a) Representative H&E staining of thoracic aorta cross‐sections showing intimal‐medial thickness and endothelial integrity (scale bar: 50 μm). (b) TUNEL staining (green) co‐localized with DAPI (blue) to detect apoptotic nuclei in vascular wall; scale bar: 20 μm. Quantification shows percentage of TUNEL^+^ cells per field. (c) Western blot analysis of Bax, Bcl‐2, and cleaved caspase‐3 in aortic lysates; GAPDH as loading control. Data are presented as mean ± SD, *n* = 3. **P < 0.01 vs. control; ^##^ *P* < 0.01 vs. model.

To investigate the effects of LIRA and DAPA, alone or in combination, on vascular endothelial dysfunction in T2DM mice, we performed histological and molecular analyses of thoracic aortic tissue. H&E staining revealed significant structural abnormalities in the model group, including marked thickening of the tunica intima and media, increased extracellular matrix deposition, and disrupted endothelial continuity (Figure 4a). In contrast, both LIRA and DAPA monotherapies partially restored vascular architecture, while the combination treatment resulted in near‐normalization of arterial wall morphology, with preserved endothelial integrity and reduced medial hypertrophy. TUNEL assay was employed to assess endothelial cell apoptosis. The model group exhibited extensive TUNEL‐positive nuclei within the vascular wall, indicating severe endothelial cell death (Figure 4b). Treatment with either LIRA or DAPA significantly reduced the number of apoptotic cells, and their co‐administration produced the most pronounced protective effect, resulting in minimal TUNEL signal comparable to that observed in the control group. Western blot analysis further confirmed the anti‐apoptotic effects of the treatments at the molecular level (Figure 4c). The model group displayed upregulated expression of pro‐apoptotic Bax and cleaved caspase‐3 and downregulated expression of anti‐apoptotic BCl‐2, consistent with enhanced mitochondrial apoptosis in diabetic vasculature. Both LIRA and DAPA effectively reversed these changes, restoring the Bax/BCl‐2 ratio and reducing caspase‐3 activation. Notably, the combination therapy yielded the most robust modulation of these markers, with Bax and cleaved caspase‐3 levels significantly lower than those in either monotherapy group, and BCl‐2 expression restored to near‐control levels. Collectively, these findings indicate that LIRA and DAPA, especially when administered in combination, mitigate T2DM‐induced endothelial dysfunction by preserving vascular integrity and attenuating endothelial cell apoptosis via modulation of the intrinsic apoptotic pathway.

### The combination enhances endothelial integrity and activates NRF2/HO‐1 and AMPK/ PKA‐eNOS signaling to counteract vascular oxidative stress in diabetes

**Figure 5:**
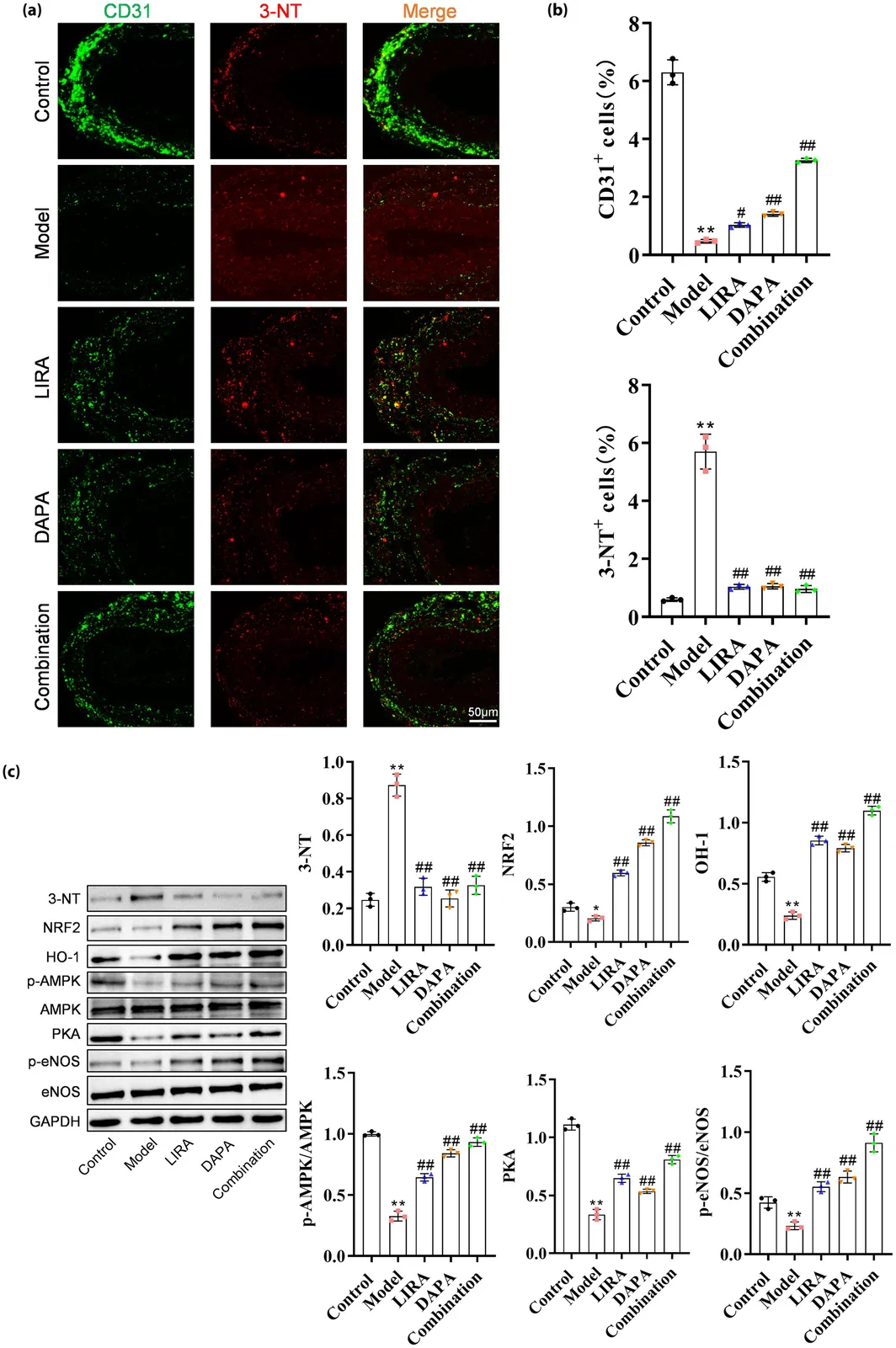
Combination therapy enhances endothelial integrity and activates NRF2/HO‐1 and AMPK/PKA‐eNOS pathways to counteract vascular oxidative stress. (a) Immunofluorescence staining of aortic sections for CD31 (endothelial marker, red) and 3‐nitrotyrosine (3‐NT, oxidative damage marker, green); nuclei stained with DAPI (blue). Scale bar: 50 μm. (b) Quantification of CD31^+^ area (%) and 3‐NT fluorescence intensity (*n* = 6 per group). (c) Western blot analysis of NRF2, HO‐1, phospho‐AMPK (Thr172), total AMPK, PKA substrate phosphorylation, eNOS, and phospho‐eNOS (Ser1177) in aortic tissue. β‐Actin served as loading control, *n* = 3. Data are presented as mean ± SD. *P < 0.05, **P < 0.01 vs. control; ^##^ *P* < 0.01 vs. model.

To elucidate the molecular mechanisms underlying the improvement of endothelial function by LIRA and DAPA, we examined oxidative stress and vascular signaling pathways in thoracic aortic tissue. Immunofluorescence staining revealed a significant reduction in CD31^+^ endothelial cells in the model group compared with controls, indicating endothelial damage and impaired endothelial integrity (Figure 5a,b). Concurrently, 3‐NT—a marker of protein nitration induced by peroxynitrite—was markedly upregulated in diabetic vessels, suggesting excessive RNS production. Both LIRA and DAPA monotherapies partially restored CD31 expression and reduced 3‐NT levels, while their combination treatment yielded the most robust effect, significantly increasing endothelial cell density and suppressing oxidative modification. Western blot analysis further confirmed the antioxidative effects of the treatments (Figure 5c). The model group exhibited elevated protein levels of 3‐NT and suppressed expression of key antioxidant regulators, including NRF2, HO‐1, and AMPK phosphorylation. Notably, both LIRA and DAPA significantly upregulated NRF2, HO‐1, and p‐AMPK levels, with the combination regimen achieving greater activation than either agent alone. Additionally, DAPA treatment enhanced PKA activity, which was further amplified in the combination group. eNOS function is critical for vascular homeostasis. We observed that eNOS protein expression was downregulated in the model group, accompanied by decreased phosphorylation at Ser1177 (p‐eNOS), a key activation site. Treatment with LIRA or DAPA restored p‐eNOS levels, and the combination therapy led to the most pronounced increase in the p‐eNOS/eNOS ratio, suggesting enhanced NO bioavailability and improved vasodilatory capacity. Collectively, these results demonstrate that LIRA and DAPA, particularly in combination, mitigate oxidative stress in the diabetic vasculature by activating the NRF2/HO‐1 and AMPK/PKA signaling pathways, restoring eNOS activity, and preserving endothelial integrity. These effects collectively contribute to the amelioration of endothelial dysfunction in T2DM.

## DISCUSSION

This study systematically evaluated the synergistic therapeutic potential of combined therapy with the GLP‐1RAs LIRA and the SGLT2i DAPA in a murine model of T2DM–induced cardiomyopathy and vasculopathy. Our key findings demonstrate that, compared with monotherapies, the combined administration of LIRA and DAPA elicits markedly enhanced cardioprotective and vasoprotective effects. These benefits not only robustly ameliorate systemic metabolic disturbances but also exert additive therapeutic actions across multiple pathological axes. Specifically, the combination therapy significantly attenuated myocardial injury and fibrosis while concurrently inhibiting aortic remodeling and endothelial cell apoptosis. Mechanistically, the superior efficacy of the dual regimen stems from its coordinated activation of the NRF2/HO‐1 antioxidative pathway and the AMPK/PKA‐eNOS vasoregulatory axis, leading to a more comprehensive mitigation of oxidative stress, inflammatory response, and mitochondrial‐dependent apoptosis in diabetic cardiovascular tissues. Collectively, these insights provide novel and mechanistically grounded preclinical evidence supporting the clinical combination of GLP‐1RAs and SGLT2is for the synergistic prevention and treatment of cardiovascular complications in diabetes.

T2DM confers a significant independent risk for the development of CVD^26^, with DCM emerging as a critical, yet distinct, entity characterized by myocardial dysfunction in the absence of coronary artery disease or hypertension^27^. The pathophysiology of DCM is multifactorial, involving a complex interplay of chronic hyperglycemia, insulin resistance, lipotoxicity, and systemic inflammation, which collectively promote myocardial fibrosis, cardiomyocyte apoptosis, and microvascular dysfunction^28^, ^29^, ^30^. Based on established individual cardiovascular benefits of GLP‐1 receptor agonists and SGLT2 inhibitors demonstrated in large‐scale clinical trials^31^, and bolstered by emerging clinical evidence showing that their combination (e.g., dulaglutide and dapagliflozin) improves endothelial glycocalyx integrity as well as vascular and myocardial function in patients with T2DM and albuminuria compared to DPP‐4 inhibitors^32^, our study further elucidates the specific pathological and molecular mechanisms underlying their synergistic effects in preclinical models of diabetes‐induced end‐organ damage. Previous research has independently established that GLP‐1RAs such as LIRA mitigate experimentally induced DCM by counteracting cardiomyocyte degeneration and reducing associated fibrosis, inflammation, and vascular rarefaction through both structural and molecular pathways^33^. Meanwhile, SGLT2is like DAPA have been shown to ameliorate cardiac fibrosis in T2DM rats, primarily by suppressing endothelial‐mesenchymal transition (EndMT) and fibroblast activation via modulation of the AMPKα/TGF‐β/Smad signaling axis^34^. However, evidence directly comparing their combined effect on the integrated cardiovascular axis—encompassing myocardial injury, fibrosis, vascular remodeling, and the crosstalk between associated signaling pathways—has been sparse.

Furthermore, the synergistic cardiovascular protection observed with the liraglutide and dapagliflozin combination appears mechanistically distinct from other common glucose‐lowering regimens. Compared to the widely used “SGLT2i + metformin” combination, which primarily augments metabolic control and insulin sensitivity^35^, ^36^, our investigated combination offers direct and complementary anti‐fibrotic, anti‐apoptotic, and anti‐inflammatory actions. Unlike the “GLP‐1RA + insulin” strategy that may be counterbalanced by weight gain, hypoglycemia, and potential adverse vascular effects^37^, the liraglutide and dapagliflozin regimen provides multidimensional benefits without these trade‐offs. This combination constructs an intervention network targeting multiple pathological pathways: dapagliflozin contributes to direct cardioprotection via natriuresis, improved myocardial energetics, and anti‐fibrotic effects, while liraglutide preserves cellular integrity through potent anti‐inflammatory and anti‐apoptotic properties. Crucially, as validated in this study, their co‐administration synergistically activates both the AMPK/PKA‐eNOS and NRF2/HO‐1 axes, thereby achieving bidirectional amplification in improving endothelial function and enhancing antioxidant defense. This integrated, multi‐targeted mechanism underpins its superior efficacy and supports its potential translational advantage over conventional combinations for comprehensive cardiovascular protection in diabetes.

Our data bridge this gap by demonstrating that the co‐administration not only produces superior phenotypic outcomes but also mechanistically integrates two pivotal protective cascades: the antioxidative NRF2/HO‐1 axis^38^, ^39^ and the vasoprotective AMPK/PKA‐eNOS axis^40^, ^41^. This coordinated multi‐pathway activation results in a more potent suppression of the key pathological triad—oxidative stress, inflammation, and apoptosis—that drives the progression of DCM and vasculopathy. Thus, our findings delineate a novel synergistic mechanism wherein LIRA and DAPA cooperatively reinforce endothelial stability and myocardial resilience, effectively intercepting the cardiovascular vicious cycle perpetuated by diabetes. This mechanistic synergy provides a compelling preclinical rationale for dual‐pathway targeting in diabetic cardiovascular complications. Echocardiographic results provide direct evidence of improved cardiac function resulting from these interventions. In our study, the T2DM model group displayed a significant reduction in EF, reflecting marked impairment of cardiac systolic function, while LIRA or DAPA monotherapy only partially restored EF. Notably, the combination therapy fully normalized EF to levels comparable with those of non‐diabetic controls, indicating that the synergistic effects of LIRA and DAPA can robustly reverse diabetes‐induced cardiac dysfunction. Consistent with the enhancements in global cardiac function, M‐mode echocardiographic images revealed that combination therapy more effectively preserved left ventricular wall thickness and contractility, underscoring its capacity to ameliorate both structural and functional deterioration in the diabetic myocardium. Importantly, our observation that combined therapy normalizes multiple serum cardiac injury markers (CK‐MB and LDH) and restores myocardial and vascular ultrastructure suggests its potential not only to attenuate but also to reverse established diabetic organ damage. These results position the LIRA/DAPA combination as a multidimensional therapeutic strategy, simultaneously addressing metabolic dysregulation, oxidative‐inflammatory burden, and cellular apoptosis across cardiac and vascular compartments.

A particularly salient implication of our findings lies in the demonstration of a coordinated cardiovascular crosstalk mechanism potentiated by the drug combination. Our data extend beyond cataloging additive benefits to suggest that the synergistic protection stems from disrupting the deleterious interplay between myocardial and vascular injury in diabetes. We propose that the robust activation of the AMPK/PKA‐eNOS axis in the vasculature does more than just improve endothelial function locally. By enhancing bioavailable NO and promoting vasodilation, it likely ameliorates coronary microvascular dysfunction^35^—a critical early event in DCM^42^—thereby improving myocardial perfusion and reducing ischemic stress on cardiomyocytes^43^. Concurrently, the amplified activation of the NRF2/HO‐1 antioxidative pathway in both cardiac and vascular tissues establishes a systemic defense barrier against the oxidative insult that fuels inflammation and apoptosis in both compartments^44^. This dual‐targeting strategy effectively intercepts the vicious cycle where vascular‐derived ROS exacerbate cardiac injury^45^, and vice versa, cardiac‐derived inflammatory mediators accelerate endothelial dysfunction^46^. Consistent with prior reports on the cardioprotective effects of dapagliflozin and/or liraglutide in models of T1DM^47^, our findings in a T2DM context similarly point to the mitigation of oxidative stress and the downregulation of inflammatory and apoptotic pathways as central mechanisms. Our data reveal that the combination therapy most effectively preserved endothelial integrity and attenuated oxidative stress in the thoracoabdominal aorta. This focus on aortic pathology is mechanistically significant, as improvements in this central artery's function and structure likely contributed to the observed cardioprotection by reducing cardiac afterload, improving coronary perfusion, and mitigating systemic oxidative‐inflammatory crosstalk. Therefore, the combined administration of LIRA and DAPA not only exerts distinct effects on the heart and vasculature, respectively, but also promotes integrated regulation of the cardiovascular system as a whole. By synergistically restoring the functional coupling between the heart and blood vessels, this combination therapy achieves a comprehensive therapeutic efficacy that surpasses the effects observed with either agent alone.

Several limitations of this study warrant consideration. First, while the HFD/STZ‐induced murine model recapitulates key features of human T2DM and its cardiac complications, it does not fully mirror the chronic, multifactorial progression of DCM and atherosclerosis in patients. Second, although we identified the activation of NRF2/HO‐1 and AMPK/PKA‐eNOS as central events, the precise upstream initiating signals triggered by each drug and the detailed molecular crosstalk between these pathways remain to be fully delineated. For instance, whether the observed synergy arises from simultaneous receptor engagement or sequential modulation of interconnected metabolic sensors requires further investigation. Third, our study employed a fixed‐dose and fixed‐duration treatment regimen. Future studies assessing dose–response relationships and long‐term outcomes, particularly in models with established heart failure, are necessary to optimize translational relevance. Lastly, although there are interspecies differences between mice and humans, large‐scale clinical trials have confirmed that both drugs (liraglutide as demonstrated in the LEADER trial^48^, ^49^ and dapagliflozin as demonstrated in the DECLARE‐TIMI 58 trial^50^, ^51^) have clear cardiovascular benefits in high‐risk patients with T2DM. Therefore, the mechanistic pathways identified in our mouse model have translational value for clinical practice. As a preclinical investigation, our findings necessitate validation in clinical studies specifically designed to evaluate intermediate cardiovascular endpoints and biomarkers of oxidative stress and inflammation in patients receiving such combination therapy.

## CONCLUSION

In conclusion, this study demonstrates that combination therapy with liraglutide and dapagliflozin confers significantly greater cardioprotective and vasoprotective benefits in a T2DM model compared to either agent administered alone. This improved efficacy appears to be attributable to the synergistic activation of the NRF2/HO‐1 and AMPK/PKA‐eNOS signaling pathways, which collectively exert more pronounced inhibitory effects on oxidative stress, inflammation, and apoptosis associated with diabetic cardiovascular injury. Collectively, these findings advance the conceptualization of liraglutide and dapagliflozin combination therapy from an empirical approach to a mechanistically grounded clinical strategy, thereby providing a robust scientific rationale for its further exploration in clinical settings.

## DISCLOSURE

The authors declare no conflict of interest.

Approval of the research protocol: All procedures involving animals were reviewed and approved by the Institutional Animal Care and Use Committee of the Kunming University of Science and Technology (KUST) (Animal protocol number: AP‐KUST‐202508020067).

Informed Consent: N/A.

Approval date of Registry and the Registration No. of the study/trial: N/A.

Animal studies: N/A.

## Data Availability

The data that support the findings of this study are available from the corresponding author upon reasonable request.
